# Regulation of Proliferation by a Mitochondrial Potassium Channel in Pancreatic Ductal Adenocarcinoma Cells

**DOI:** 10.3389/fonc.2017.00239

**Published:** 2017-09-29

**Authors:** Roberta Peruzzo, Andrea Mattarei, Matteo Romio, Cristina Paradisi, Mario Zoratti, Ildikò Szabò, Luigi Leanza

**Affiliations:** ^1^Department of Biology, University of Padova, Padova, Italy; ^2^Department of Pharmaceutical and Pharmacological Sciences, University of Padova, Padova, Italy; ^3^Department of Chemical Sciences, University of Padova, Padova, Italy; ^4^Institute of Neuroscience, CNR, Padova, Italy; ^5^Department of Biomedical Sciences, University of Padova, Padova, Italy

**Keywords:** ion channels, mitochondria, cell cycle, ROS, pancreatic ductal adenocarcinoma

## Abstract

Previous results link the mitochondrial potassium channel Kv1.3 (mitoKv1.3) to the regulation of apoptosis. By synthesizing new, mitochondria-targeted derivatives (PAPTP and PCARBTP) of PAP-1, a specific membrane-permeant Kv1.3 inhibitor, we have recently provided evidence that both drugs acting on mitoKv1.3 are able to induce apoptosis and reduce tumor growth *in vivo* without affecting healthy tissues and cells. In the present article, by exploiting these new drugs, we addressed the question whether mitoKv1.3 contributes to the regulation of cell proliferation as well. When used at low concentrations, which do not compromise cell survival, both drugs slightly increased the percentage of cells in S phase while decreased the population at G0/G1 stage of cells from two different pancreatic ductal adenocarcinoma lines. Our data suggest that the observed modulation is related to ROS levels within the cells, opening the way to link mitochondrial ion channel function to downstream, ROS-related signaling events that might be important for cell cycle progression.

## Introduction

Pancreatic ductal adenocarcinoma (PDAC) is considered a silent killer. A large number of new cases arise every year, but the lifespan of the patients is always very short, with a survival percentage around 25% 5 years after diagnosis (1). PDAC treatment is limited prevalently to the use of fluorouracil or gemcitabine either alone or in combination and to surgery following diagnosis (2). Nowadays, novel strategies, supported by experimental observations, are emerging as possible therapeutical options. Among possible new targets, an important role has been assigned to ion channels: these proteins have been related to several hallmarks of cancer, ranging from resistance to cell death, to modifying and controlling cell cycle progression, as well as to favor tumor progression and metastasis formation (3–5). A particular role has been proposed for potassium-selective ion channels. Potassium (K^+^) channels are present in basically every organism, ranging from virus to mammals and permit K^+^ transport across biological membranes. They are formed by tetramers of four α subunits and by regulatory β and γ subunits. The selectivity filter is almost universal and is formed by a five residue-long signature sequence (TVGYG) within the pore loop of each subunit, as it has been demonstrated both by site-directed mutagenesis combined with electrophysiology and by the resolution of the crystal structure of several potassium channels (6–9). The specificity for cations and in particular K^+^ ions is due both to the presence of a set of negatively charged amino acids in the vestibule region and of carbonyl oxygen atoms in the filter region, which precisely mimic the configuration of oxygen atoms around a solvated K^+^ ion (10). The negative residues are evolutionary conserved and can interact with a preserved positively charged amino acid present in the peptide toxins of some venoms, which can block the channel (11).

Plasma membrane (PM) K^+^ channels are involved in the regulation of several fundamental cellular functions, including apoptosis and cell cycle progression. By regulating K^+^ permeability across the PM, K^+^ channels are able to change membrane potential. During G1 to S phase progression, opening of these channels leads to an increased K^+^ permeability and hyperpolarized PM, while their closure partially depolarizes the PM favoring thus M phase transition (12). The regulation of the proliferation is mediated also by control of the PM Ca^2+^ permeability *via* calcium channels through the membrane potential, that can be modulated *via* K^+^ channels. The role of PM K^+^ channels in proliferation and regulation of calcium influx has been extensively studied thanks to several impermeant specific K^+^ channels inhibitors, such as Margatoxin, Stichodactyla toxin (ShK), Charybdotoxin, etc. Block of PM K^+^ channels by these small peptide inhibitors generally results in reduced Ca^2+^ influx and block of the cell cycle and cellular proliferation [e.g., Ref. (13, 14)].

Robust experimental evidence indicates that intracellular counterparts of the PM-located K^+^ channels exist in different membranes such as Golgi, endoplasmic reticulum, nucleus, lysosomes, and mitochondria (15, 16). In some cases, especially in that of mitochondrial channels, an important role for cancer cell development and progression is emerging (17). In collaboration with the groups of Professors Gulbins and Kalthoff, we have recently demonstrated that pharmacological targeting of a mitochondrial K^+^ channel, namely of Kv1.3 of the shaker family (mitoKv1.3), efficiently triggers programmed cell death (18) and provides a new tool to selectively eliminate cancer cells even *in vivo* (19, 20). In an orthotopic mouse PDAC model using Colo357 cells, three membrane permeant Kv1.3 inhibitors, namely Psora-4, PAP-1, and clofazimine, led to cancer cell death *in vitro*. *In vivo*, a reduction of the tumor weight by approximately 50% occurred when using clofazimine, without inducing side effects on healthy cells and organs (20). Very recently, we have developed a new class of mitochondria-targeted Kv1.3 inhibitors starting from the PAP-1 molecule (19). A positively charged triphenylphosphonium moiety was added to PAP-1 either directly (PAPTP) or *via* a carbamoyl linker (PCARBTP) to allow a preferential targeting of the molecule to mitochondria (characterized by approximately −180 mV membrane potential that drives accumulation of the positively charged PAP derivatives) and thus, a direct effect of these new Kv1.3 inhibitors on the mitochondrial channels. These results demonstrated that the PAP-1 derivatives are more efficient than their precursors in killing various types of cancer cells in *in vitro, ex vivo*, and *in vivo* experiments. Although apoptotic cells were observed in the tumor tissue, the question remained open whether alteration of the function of the mitoKv1.3 might impact tumor volume, not only by inducing apoptosis at high concentrations, but also by altering cell proliferation at sublethal concentrations.

In the present article, we investigated the possibility that these new compounds, used at low concentrations, alter cell cycle either by acting on the PM Kv1.3 channel or by acting on the mitoKv1.3 in a highly metastatic PDAC cell line.

## Materials and Methods

### Cell Culture

PANC-1 cell line was routinely grown in Dulbecco’s modified Eagle’s medium (DMEM) supplemented with 10% fetal bovine serum, 10 mM HEPES (pH 7.4), 100 µM non-essential amino acids, 100 U/ml penicillin, 100 µg/ml streptomycin (all Life Technologies) in a humidified atmosphere with 5% CO_2_ at 37°C. Colo357 cells were maintained in RPMI medium supplemented as stated before for DMEM.

### Reagents

All membrane-permeant substances were protected from UV sources to prevent their photo-oxidation. Psoralen, 5-(4-Phenoxybutoxy) psoralen (PAP-1; Merck-Sigma-Aldrich, Germany), PAPTP, PCARBTP, clofazimine (Merck-Sigma-Aldrich, Germany) were dissolved in dimethyl sulfoxide (DMSO). Staurosporine (Merck-Sigma-Aldrich, Germany) was dissolved in absolute ethanol (EtOH), and diluted in DMEM. The final concentration of DMSO was ≤0.5% in all assays.

### MTS Assay

To measure viability of the cells, we used the tetrazolium reduction (MTS) assay. Cells were seeded into 96-well plates at a density of 5 × 10^3^ cells/well and allowed to grow in DMEM (supplemented as described before) for 24 h. The growth medium was then replaced with phenol red and FBS-free medium and treated with the drugs at increasing concentrations: four wells were used for each condition. After 24 h 10% CellTiter 96^®^ AQUEOUS One solution (Promega, Italy) was added to each well as indicated by the supplier. 4 h after incubation at 37°C, absorbance at 490 nm was measured using an Infinite^®^ 200 PRO 96-well plate reader.

### Western Blotting

Cells (1 × 10^6^) were trypsinized and centrifuged at 500 *g* for 10 min. The pellet was then resuspended in 300 µl of lysis buffer (25 mM TRIS pH 7.8, 2.5 mM EDTA, 10% glycerol, 1% NP40, 2 mM DTT), frozen at −80°C, thawed and then vortexed for 10 sec. Samples were centrifuged at 20,000 *g* for 10 min at 4°C. To enhance protein separation, supernatant samples were solubilized for 1 h at RT in Sample Buffer (SB: 30% glycerol + 125 mM Tris pH 6.8 + 9% SDS + 0.1 M DTT + 0.3% bromophenol blue), loaded on SDS-PAGE (10% polyacrylamide gel, 15–25 mV). After separation by electrophoresis, gels were blotted overnight at 4°C onto PVDF membranes. After blocking with a 10% solution of defatted milk, the membranes were incubated overnight at 4°C with the following primary antibodies: anti-Kv1.3 (1:200, rabbit polyclonal; Alomone Labs APC-101); anti-GAPDH (1:1,000, mouse monoclonal; Millipore MAB374). After washing, the membranes were developed using corresponding antimouse or antirabbit secondary antibodies (Calbiochem). Antibody signal was detected with enhanced chemiluminescent substrate (SuperSignal West Pico Chemiluminescent Substrate; Thermo Scientific).

### ROS Production

PANC-1 cells (1 × 10^6^ cells for each condition) were incubated for 20 min at 37°C in the dark in DMEM medium without serum and phenol red with 1 µM Mitosox (Thermo Scientific) or 2 µM dihydroethidium (DHE). In some conditions, cells were preincubated for 1 h with ROS scavengers, Mitotempo, and N-acetylcysteine (NAC). Cells were treated with the compounds for 2 h and the fluorescence of the probes was normalized with the basal fluorescence measure before drugs addition by FACS (FACSantoII, BD Bioscience).

### Cell Cycle Analyses by Flow Cytometry

PANC-1 and Colo357 cells were plated in 6-well plates at a density of 1 × 10^5^ and 5 × 10^5^ cells/well plates, respectively. The following day, cells were treated with different Kv1.3 inhibitors at sublethal concentrations. After 24 h, cells were collected, washed in cold PBS, permeabilized in 70% EtOH, and then stored at 4°C. After 48 h, cells were centrifuged, washed in cold PBS, centrifuged again, resuspended in the staining mix (constituted by PBS, propidium iodide PI; 50 µg/ml; Merck-Sigma-Aldrich) and RNaseA (10 µg/ml; Qiagen), and incubated for 1 h at 37°C. Data were acquired using FACSanto II (BD Biosciences) and analyzed through ModFit LT software.

### Apoptosis Analyses Using Fluorescence Microscopy

PANC-1 cells were seeded at a density of 4 × 10^4^ cells/well in 24-well plate. The following day, the medium of each well was replaced with phenol red and FBS-free DMEM and treated with different Kv1.3 inhibitors. After 24 h, Annexin V-Alexa568 (Roche) was added to each well and cells were incubated for 45 min at 37°C. Images were acquired using the fluorescence microscope Leica DMI 4000.

### Statistical Analyses

Experiments were repeated at least three times with consistent results. Unless otherwise stated, data are expressed as the mean ± SEM. The results were analyzed using Student’s *t*-test (for MTS and Apoptosis analysis) and Two-way ANOVA with Bonferroni’s posttest correction (for ROS and cell cycle analysis). In all cases, *p* ≤ 0.05 was considered as statistically significant difference.

## Results

### PANC-1 Cells Are Sensitive to Treatment with Kv1.3 Inhibitors

**Figure 1:**
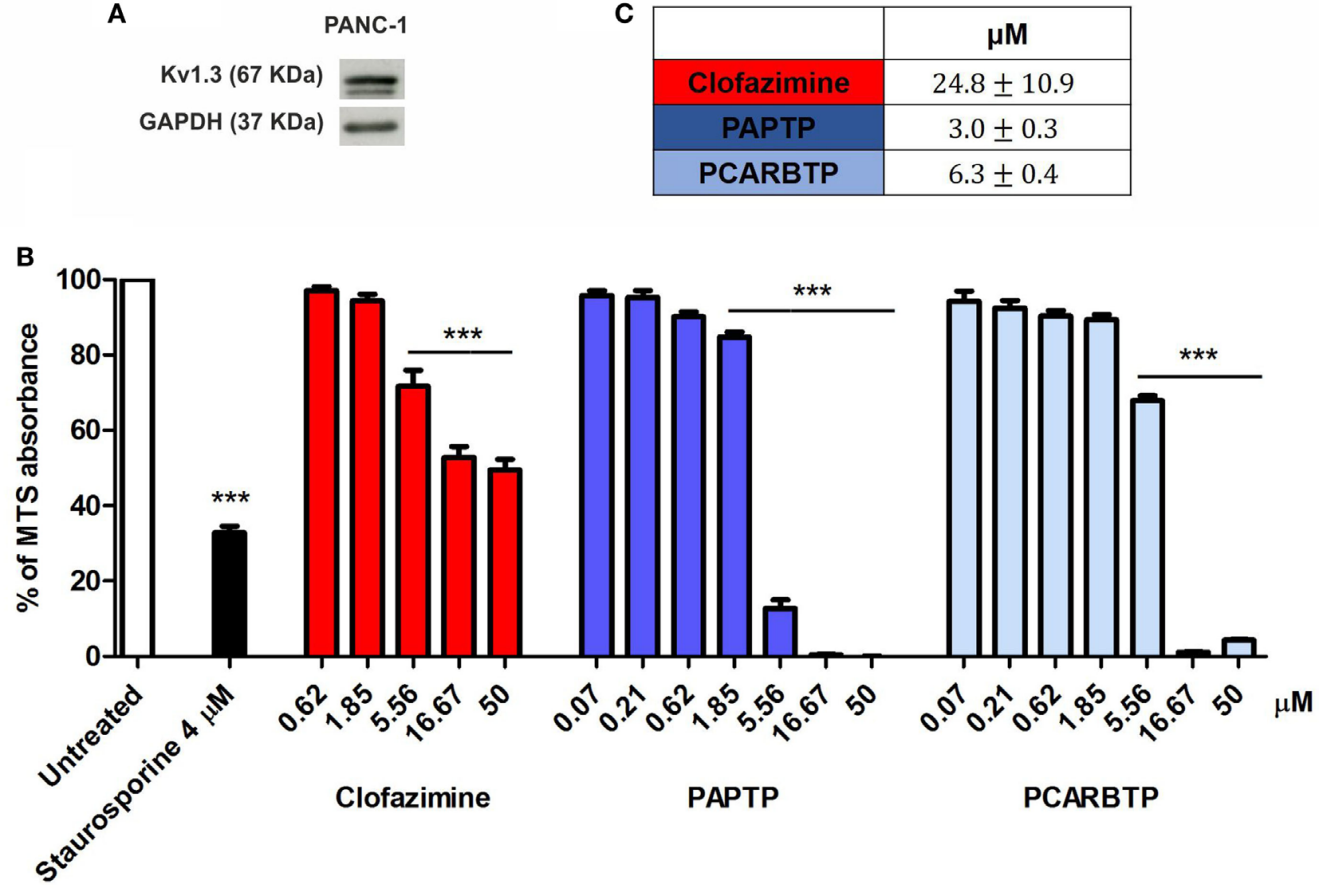
Potassium channel Kv1.3 is expressed in PANC-1 cells. **(A)** Kv1.3 expression was determined by Western Blot in PANC-1 cells. 60 µg of total protein extract were loaded into a SDS gel and blotted onto a polyvinylidene fluoride (PVDF) membrane. Kv1.3 band was evaluated by immunoblotting with a specific antibody. GAPDH was used as loading control. The blot is a representative image of three different observations. **(B)** Inhibition of mitochondrial Kv1.3 by different concentration of membrane permeant blockers resulted in a reduction of the MTS signal from PANC-1 cells. Values are reported as percentage respect to untreated sample ± SEM. All compounds were added for 24 h. Staurosporine was used as positive control (*n* = 3; ***p < 0.001). **(C)** Determination of the EC_50_ of the indicated compounds in PANC-1 cells treated as in **(B)**. EC_50_ was determined by using Origin60 software. First, a logarithmic dose response curve was generated using MTS data and then the EC_50_ values were calculated using the Origin Software.

**Figure 2:**
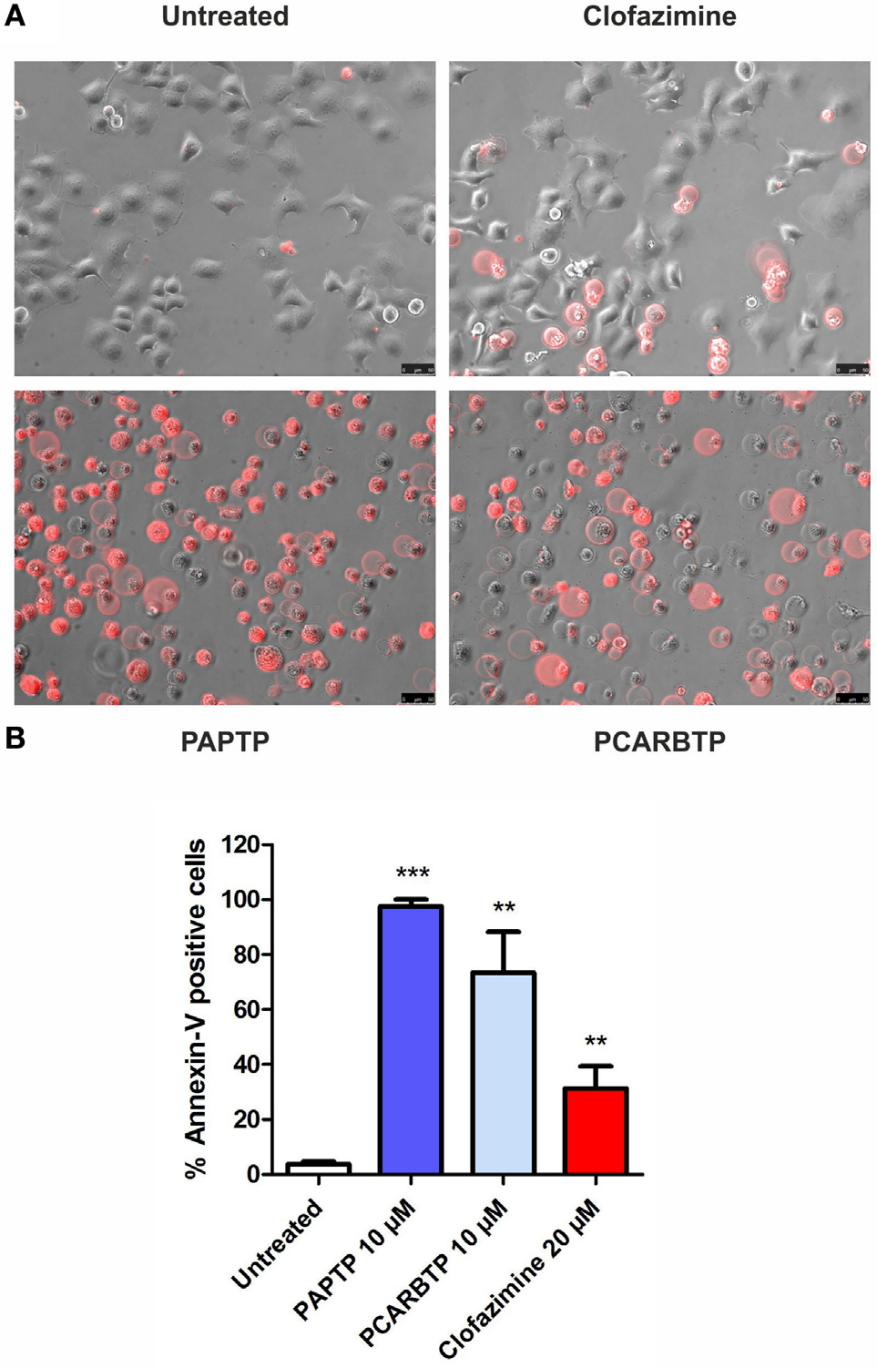
Inhibition of mitochondrial Kv1.3 kills PANC-1 cells. **(A)** PANC-1 cells were treated or left untreated with different membrane permeant Kv1.3 inhibitors (PAPTP 10 µM, PCARBTP 10 µM, and clofazimine 20 µM) for 24 h. Cell death was determined by staining with an Alexa568 coupled Annexin V by fluorescent microscopy. The images are representative of three different replicates. **(B)** Quantification of Annexin V-positive cells from the experiments shown in **(A)**: represented is the percentage of Annexin V-positive cells ± SEM (*n* = 3; **p < 0.01; ***p < 0.001).

The highly metastatic PDAC cell line PANC-1 was used since it expresses a high level of the antiapoptotic Bcl-xL (21) and the action of mitoKv1.3 inhibitors is expected to be independent of Bax/Bcl-xL/Bcl-2 ratios. We have previously demonstrated that Kv1.3 is highly expressed in several PDAC cell lines both at transcript and protein level (20). Figure 1A shows that Kv1.3 protein is expressed in PANC-1 cells. The predominant band observed for anti-Kv1.3 antibody corresponded to the protein with an apparent molecular size of 67 kDa, which is similar to the predicted size of Kv1.3 subunits deposited in the database (Kv1.3 P15384). Next, we tested sensitivity of PANC-1 cells to treatment with membrane permeant Kv1.3 inhibitors. In particular, we incubated these cells for 24 h with clofazimine or with the newly synthetized PAP-1 derivatives, PAPTP and PCARBTP (Figures 1B,C). Clofazimine, a drug already used in clinical practice to treat leprosy and some autoimmune diseases, was able to reduce cell survival with an EC_50_ of 25 µM. These cells were even more resistant to one of the most specific small molecule psoralen Kv1.3 inhibitor, PAP-1, even when applied together with CSH in order to avoid export of PAP-1 from the cells (19). Contrarily, its recently synthetized derivatives, PAPTP and PCARBTP, were more powerful and could decrease the MTS signal that is related to cell survival with an EC_50_ of approximately 3 and 6 µM, respectively (Figures 1B,C). The decrease of MTS values was due to apoptosis, as 10 µM PAPTP or PCARBTP caused a drastic increase in programmed cell death as assessed using Annexin V staining (Figure 2), while clofazimine even at 20 µM concentration induced only less than 30% cell death.

### Both Membrane Impermeant and Membrane Permeant Kv1.3 Inhibitors Affect Cell Cycle

**Figure 3:**
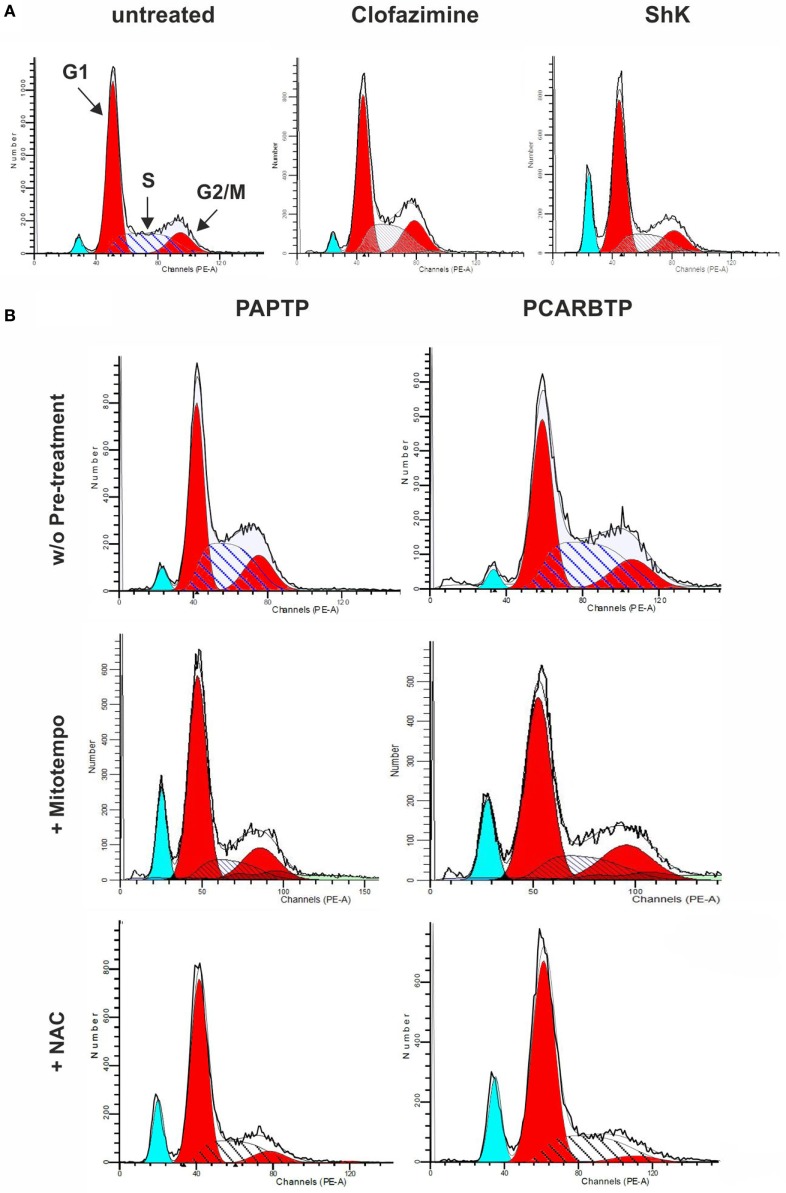
Sublethal inhibition of mitochondrial Kv1.3 leads to cell cycle alterations. **(A)** FACS analysis of cell cycle of PANC-1 cells untreated or treated with clofazimine 1 µM and ShK 100 nM for 24 h. The distribution was determined by staining cells with 50 µg/mL Propidium Iodide and the acquisition was performed by a FACSantoII (Beckton Dickson). The plots are representative of three separated determinations. Light blue peaks represent apoptotic cells, while red peaks represent cells in G1 and G2/M phases. S phase is represented by the area with blue straight lines. A sub-G1 peak is due to dead cells. In the graph of untreated cells the labels and the arrows identify the different populations that are reported. **(B)** Cell cycle distribution in PANC-1 cells pretreated (lower panels) or not (upper panels) with 50 µM Mitotempo or 20 mM N-acetylcysteine (NAC) for 1 h, before the addition of mitochondrial Kv1.3 inhibitors PAPTP and PCARBTP (both at 100 nM) for 24 h. The experiment and the analysis have been performed as in **(A)**. The plots are representative of three separate measurements.

**Figure 4:**
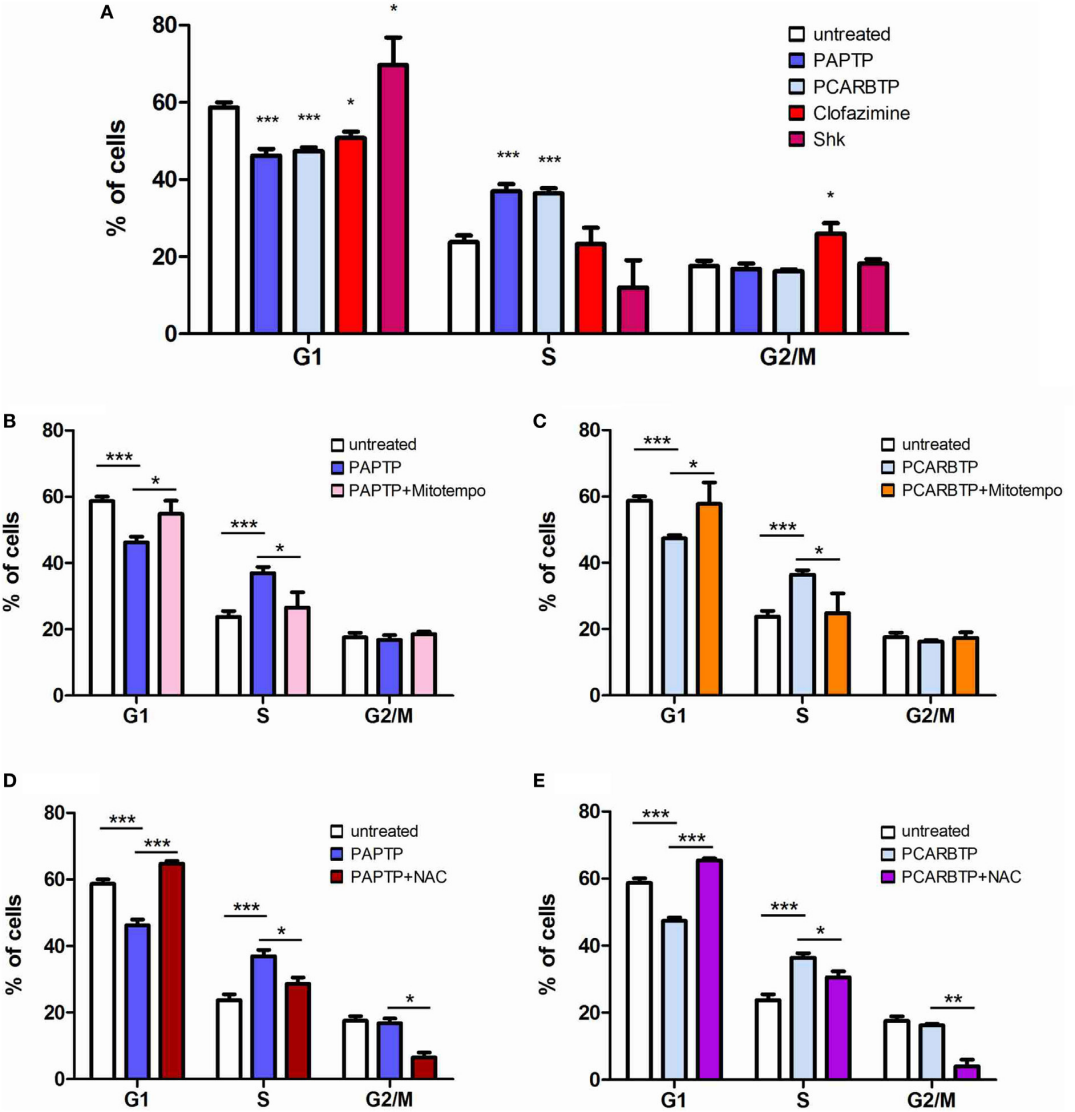
ROS favor cell cycle progression in PANC-1 cells. **(A)** Quantification of percentage of PANC-1 cells in the different phases of the cell cycle (G1, S, G2/M) in the experiments shown in Figures 3A,B. The analysis was performed by the ModFIT software. Values are reported as percentage of cells in the different phases ± SEM (*n* = 3; *p < 0.05; ***p < 0.001). **(B–E)** Quantification of the cell cycle distribution as in **(A)** of PANC-1 cells treated as in Figure 3B with PAPTP **(B,D)** or PCARBTP **(C,E)**. Values are reported as percentage of cells in the different phases ± SEM (*n* = 3; *p < 0.05; **p > 0.01; ***p < 0.001).

In order to understand whether the above Kv1.3 inhibitors affect proliferation rate and cell cycle, we used a very low, l00 nM concentration of PAPTP and PCARBTP and 1 µM clofazimine, which left all cells alive (see Figure 1B). In addition, a membrane-impermeant, highly specific Kv1.3 inhibitor, Shk (100 nM) was used in order to compare the effects of PM Kv1.3 with those of the mitochondrial counterpart. Clofazimine acts on both PM and mitoKv1.3, while the mitochondriotropic drugs act prevalently on mitoKv1.3 and Shk acts only on PM Kv1.3. After incubation, cells were rapidly fixed and their DNA was stained by propidium iodide in the presence of RNAase. Cellular DNA content was used to determine cell cycle phase of each cell by FACS analysis. Cell cycle distribution was analyzed with the ModFit software (Figures 3A,B). Representative histograms are shown in Figure 3 while Figure 4 reports statistical analysis from 4 independent replicates. While Shk diminished the percentage of cells in S phase and at the same time it increased the population in G1 phase (Figures 3A and 4A) supporting the notion that Shk reduces proliferation, a slight, proliferation-supporting effect has been observed for both PAPTP and PCARBTP. With these latter substances a slight but statistically significant decrease in G1 phase was found after treatment with the low (100 nM) doses of the mitochondriotropic compounds only, with an increase in the fraction of cells in S-phase, which is a parameter widely used to describe the proliferative capacity of the cells (Figures 3B and 4A). Interestingly, clofazimine slightly decreased the percentage of cells in G1 phase while increased the portion of cells in G2/M phase (Figures 3A and 4A).

### Membrane Permeant Mitochondriotropic Kv1.3 Inhibitors Affect Cell Proliferation *via* ROS Production

**Figure 5:**
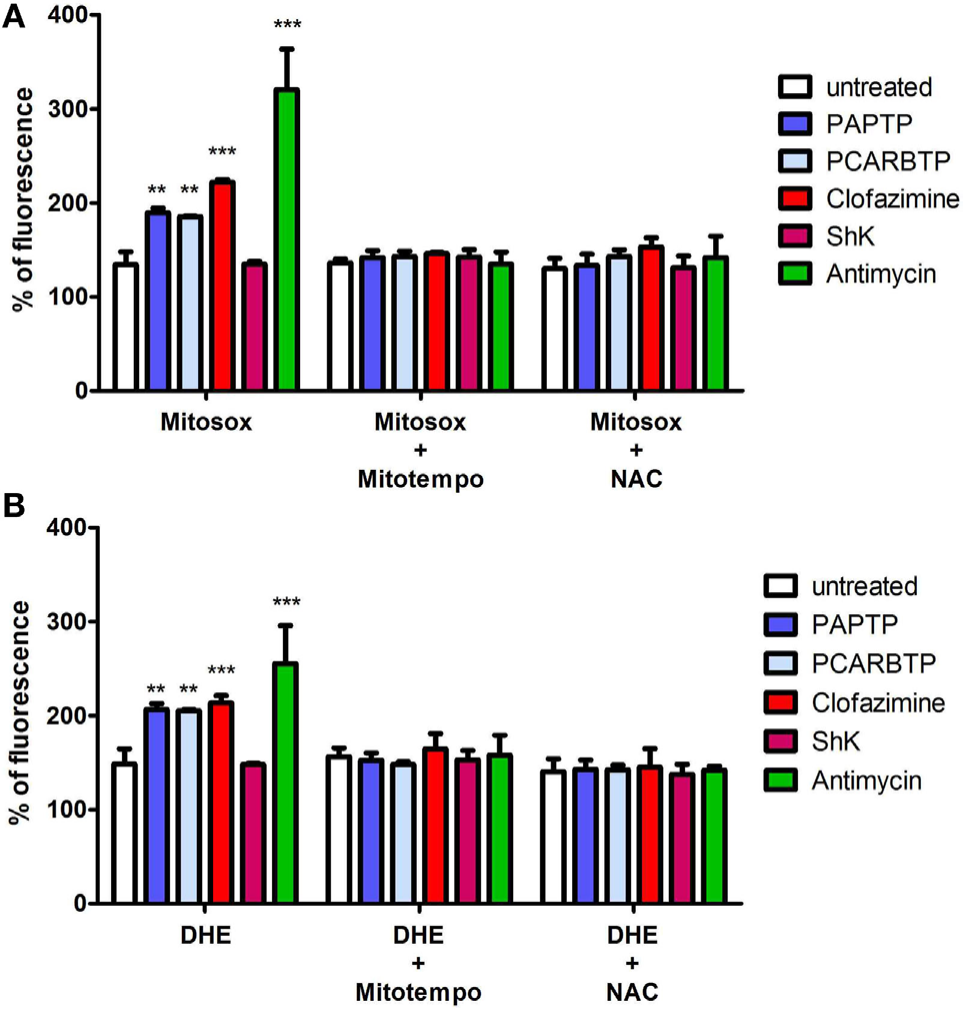
Low concentrations of PAP-1 derivatives induces ROS production. **(A)** Quantification of mitochondrial ROS production in PANC-1 cells after 2 h of incubation with PAPTP, PCARBTP, and ShK (all 100 nM), Antimycin A (2 µM) or clofazimine (1 µM) by Mitosox (1 µM). Where indicated cells were pre-treated with ROS scavengers Mitotempo (50 µM) or N-acetylcysteine (NAC) (20 mM) for 1 h. Values are reported as percentage of Mitosox fluorescence compare to 0 h ± SEM (*n* = 3; **p > 0.01; ***p < 0.001). **(B)** Quantification of intracellular ROS production in PANC-1 cells treated as in **(A)** after 2 h by DHE (1 µM). Values are reported as percentage of DHE fluorescence compare to 0 h ± SEM (*n* = 3; **p > 0.01; ***p < 0.001).

Mild oxidative stress has been linked to increased proliferation (22, 23) and we have previously shown that both mitochondriotropic drugs, PAPTP and PCARBTP, when used at concentrations that kill the cells, are able to substantially increase ROS release at the level of mitochondria (19). ROS production occurs because inhibition of mitoKv1.3 results in hyperpolarization and changes in the mitochondrial membrane potential increase the likelihood of electron transfer to molecular oxygen mainly at the level of respiratory chain complexes I and III, e.g., Ref. (24, 25). We hypothesized that sublethal, low concentrations of these drugs may induce only a mild oxidative stress to the cells, thereby promoting proliferation. To prove this hypothesis, we measured both mitochondrial and intracellular ROS production in PANC-1 cells after 2 h of incubation with our compounds (Figures 5A,B). Statistically significant increase of ROS release in the mitochondria as well as of ROS level in the cytosol was observed. This increase was completely prevented by pre-incubating PANC-1 cells with ROS scavengers, either the mitochondriotropic Mitotempo or the antioxidant NAC. Both Mitotempo and NAC were used at a concentration that had previously been shown to be effective in different cell lines to scavenge mitochondrial ROS (19, 26). To further prove the contribution of ROS to cell cycle progression, we measured the cell cycle parameters both in untreated and in treated cells, as well as in those preincubated with ROS scavengers (Figures 4B–E). Figure 3B shows representative histograms, while Figures 4B–E demonstrates that in the case of both Kv1.3-affecting drugs, both Mitotempo and NAC reverted the effects of the mitochondriotropic drugs on cell cycle in a statistically significant way. Interestingly, the mitoKv1.3 inhibitors induced a decrease of the cell population in G2/M phase when applied following NAC preincubation, possibly due to some additional effects of NAC with respect to Mitotempo.

**Figure 6:**
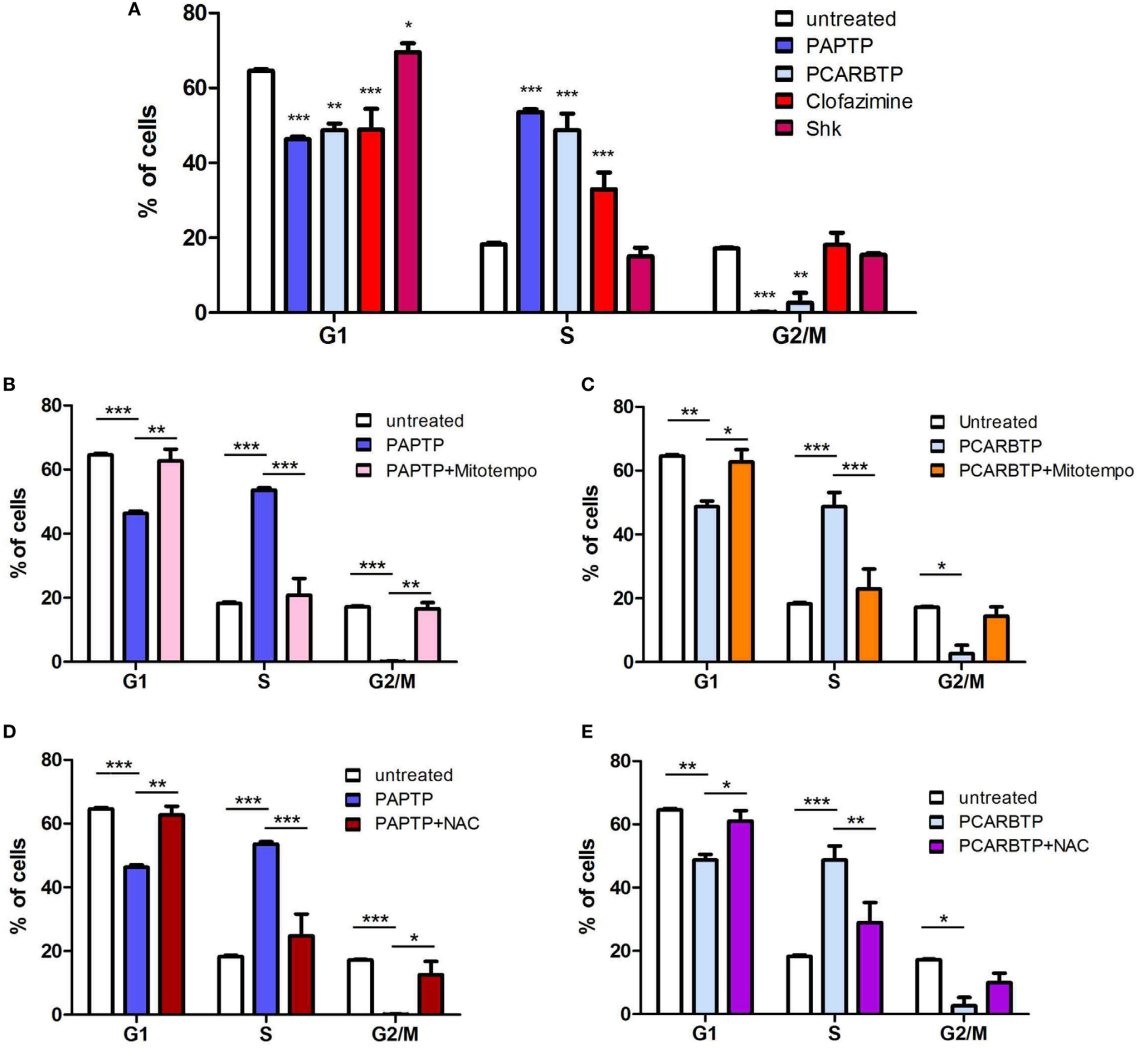
ROS favor cell cycle progression in Colo357 cells. **(A)** Quantification of percentage of Colo357 cells in the different phases of the cell cycle (G1, S, G2/M) in the experiments shown in Figures 3A,B. The analysis was performed by the ModFIT software. Values are reported as percentage of cells in the different phases ± SEM (*n* = 3; *p < 0.05; **p > 0.01; ***p < 0.001). **(B–E)** Quantification of the cell cycle distribution as in **(A)** of Colo357 cells treated as in Figure 3B with PAPTP **(B,D)** or PCARBTP **(C,E)**. Values are reported as percentage of cells in the different phases ± SEM (*n* = 3; *p < 0.05; **p > 0.01; ***p < 0.001).

To consolidate these observations, we repeated these experiments using another metastatic PDAC cell line, namely Colo357. These cells underwent cell death after treatment with high concentrations of these compounds, but a different EC_50_ compared to PANC-1 has been obtained [3.7, 2, and 1.5 µM for PAPTP, PCARBTP, and clofazimine, respectively (19, 20)]. We incubated Colo357 cells with sub-lethal doses of these drugs. The concentrations to be used were calculated keeping the same proportion of the drug concentration with respect to the EC_50_ that we used for PANC-1 (i.e., 30–60 times lower concentration than the respective EC_50_ values; PAPTP 100 nM; PCARBTP 35 nM; clofazimine 40 nM) (Figures 6A–E). As shown in Figure 6, Colo357 cells behaved similar to PANC-1 in the regulation of their cell cycle. Interestingly, in this cell line both PAPTP and PCARBTP caused not only an increase of the cell population in the S phase, but also a drastic decrease in the G2/M phase.

## Discussion

In this work, we assessed the effect of the recently described mitochondria-targeted Kv1.3 inhibitors on cell cycle in two different PDAC cell lines, when used at very low concentrations that did not affect cell death. Kv1.3 is highly expressed in the PM of various cells (3, 5). Elevated Kv1.3 expression is detected in a number of human malignancies (27) including breast, colon, and prostate cancer, leukemia (28), melanoma (18), and PDAC (20) Robust evidence demonstrates that Kv1.3 regulates proliferation and cell cycle progression (13), *via* modulation of the PM potential, a key regulator of proliferation in a number of cell types. Modulation of the membrane potential is required for both G1/S phase and G2/M phase transitions: during G1/S the cell membrane becomes hyperpolarized relative to the resting potential as voltage-gated Kv potassium channels mediate the efflux of positively charged K^+^ ions from the cells to the extracellular milieu, e.g., Ref. (29, 30); conversely, depolarization of the PM seems to be essential for the G2/M transition. In B cell lymphocytes, inhibition of K^+^ channels induced a reversible cycle arrest (31). Knockdown or inhibition of Kv1.3 and Kv1.5 in rat oligodendrocytes resulted in cell cycle arrest at G1/S (32, 33). Specific Kv1.3 blockers that act exclusively on PM Kv1.3, such as Margatoxin or Shk and its analogues have also been reported to block proliferation of T lymphocytes (34), of rat prostate cancer cells (35), of human endothelial cells (36) and of oligodendrocyte progenitor cells (32). Our results obtained using Shk are compatible with those observed for Margatoxin on the cell cycle, as reported for A549 human lung cancer cells (37): both molecules slightly decrease the percentage of cells in the S phase, while increasing the population in G0/G1 phase, indicating a block at the G1/S transition. K^+^ channel activities regulate G1 progression in the cell cycle also by modulating the activity of some of the crucial proteins involved in G1–S phase progression, including cyclin or cyclin-dependent kinase inhibitors (cdki) (33) like members of two distinct cdki families, INK4 and Kip/CIP. It has been demonstrated for example in lung cancer A549-cultured cells that the antiproliferative effect of Margatoxin or of silencing of Kv1.3 expression is mediated *via* G1-S transition block *via* a mechanism that involves p21Waf1/Cip1 accumulation and decrease of Cdk4 and cyclin D3 (37). Cyclin D3, Cdk4, and p21Waf1/Cip1 are important factors that determine the regulation of the G1 phase progression of the cell cycle (38): Cyclin D binds to Cdk4 and p21Waf1/Cip1 inhibits the cyclin D/Cdk complexes. Similarly, p21 and p27 accumulation has been reported to account for cell cycle arrest at G1 in rat oligodendrocyte precursors (32). As to clofazimine, it has been proposed to block proliferation by phospholipase A2-mediated mechanisms (39) and its application was shown to slightly reduce proliferation of Colo357 cells in a PDAC *in vivo* model, as evaluated by using anti-Ki67 antibodies on tumor tissues (20). Ki67 is a nuclear protein which is highly expressed in particular during late G1-S-M and G2 phases of the cell cycle. However, clofazimine may exert its action at various levels (40). Interestingly, the effect of clofazimine, i.e., and increase of the cell population in G2/M phase is similar to that obtained using a lentiviral-dominant negative approach to obtain loss of function of Kv1.3: this latter approach was shown to mediate reversion of effector memory T cells into central memory T cells *via* a delay in cell cycle progression at the G2/M stage. The inhibition of Kv1.3 signaling caused an increase of SMAD3 phosphorylation and induction of nuclear p21cip1 with consequent suppression of cyclin-dependent kinase 1 (Cdk1) and cyclin B1 (41). PM Kv1.3 was also shown to play an important role in cell cycle activation by controlling Akt phosphorylation (42). Interestingly, Kv1.3 seems to harbor also some functional roles independently of its ability to form potassium channels as expressing a non-conducting mutant of Kv1.3 (43) was demonstrated to be sufficient in promoting allografted tumor growth.

In sharp contrast with the effect of Shk and clofazimine on cell cycle progression, the mitochondriotropic Kv1.3 inhibitors slightly increased proliferation when used at low concentrations, as indicated by the increase and decrease of the percentage of cells in S phase and G0/G1 phase, respectively. On the other hand, the same drugs seems to block proliferation of Colo357 cells, by preventing progression from S phase to the G2/M phase (44). Although knock-down of VDAC1 was shown to reduce proliferation in HeLa cells (45), for the major part of mitochondrial ion channels, their possible involvement in the regulation of cell cycle progression has not been addressed, to our best knowledge. This may be due to either the lack of specific inhibitors or to the lack of the molecular identity of the channel proteins, preventing thus the use of genetic models (46). The mechanism leading to a slight increase of proliferation upon inhibition of mitoKv1.3 might involve different downstream events. Since these drugs induce a mild ROS release (Figure 5) even at low concentrations (19), and a slight oxidative stress may favor cell survival and proliferation (22, 23, 47), the observed effect is mediated by ROS produced by mitochondria upon inhibition of Kv1.3. The results shown in Figures 3, 4 and 6, that clearly indicate the reversal of the effects of the drugs upon pre-incubation of the cells with a mitochondria-targeted ROS scavenger, strongly supports the above hypothesis. Through the mechanism of cysteine oxidation, ROS, in particular H_2_O_2_, can modify protein structure and function in order to influence signaling cascades (22). For example, H_2_O_2_ inhibits the phosphatase Cdc14B, allowing activation of Cdk1, necessary to drive cell cycle progression. Mitochondria-released ROS was shown to enhance phosphoinositide 3-kinase signaling, which in turn leads to activation of Akt, driving proliferation (48). Other phosphatases that are known to be inhibited by ROS are protein tyrosine phosphatase PTP1b and MAPK phosphatases, which also regulate pathways critical for cellular proliferation (49). In addition, ROS has been shown to directly target certain kinases and transcription factors. Future work will clarify the downstream ROS-related (and possible ROS-unrelated) signaling events leading to alterations of cell cycle upon application of PAPTP and PCABTP. The present work opens the possibility to investigate in detail the plethora of possible pathways leading to increased proliferation by specific modulation of the function of a mitochondrial ion channel. The question also arises whether the mild effect of the new PAP-1 derivatives observed at very low concentrations might compromise their efficiency *in vivo*. Please note that at the concentrations used, PAPTP and PCARBTP induce a rapid apoptosis of the cells, even *in vivo* as observed in tumoral tissues after treatment (19). However, it cannot be excluded that in the case of PDAC where stroma may impede the homogenous diffusion of the drugs in general within the tumor mass, certain zones of the solid tumor might experience a low, sub-lethal concentration of PAPTP and or PCARBTP. While further work will be necessary to establish whether an increase of Ki67 index can occur at least in some parts of the tumor mass *in vivo* in PDAC models, this issue may not apply to hematopoietic malignancies and to more easily penetrable solid tumors such as melanoma.

## Author Contributions

RP, LL, AM, and MR: performed experiments. LL, IS, MZ, and CP: designed research. LL, IS, MZ, and CP: analyzed results. LL and IS: wrote the article.

## Conflict of Interest Statement

The authors declare that the research was conducted in the absence of any commercial or financial relationships that could be construed as a potential conflict of interest.
